# Does sensation-seeking behavior influence the patterns of flavored e-cigarette use? A cross-sectional study among Indonesian adolescents and young adults

**DOI:** 10.1186/s12889-024-18626-3

**Published:** 2024-04-24

**Authors:** Mouhamad Bigwanto, Melinda Pénzes, Róbert Urbán

**Affiliations:** 1 Doctoral School of Psychology, Eötvös Loránd University, Izabella U. 46, Budapest, 1064 Hungary; 2 Institute of Psychology, Eötvös Loránd University, Izabella U. 46, Budapest, 1064 Hungary; 3 Faculty of Health Sciences, Universitas Muhammadiyah Prof. Dr. HAMKA, Jl Limau II, Jakarta, 12210 Indonesia; 4 Data-Driven Health Division of the National Laboratory for Health Security, Health Services Management Training Centre, Semmelweis University, Kútvölgyi út 2, Budapest, H-1125 Hungary

**Keywords:** Electronic cigarettes, Flavor, Sensation-seeking behavior, Adolescent, Young adult, Student, Indonesia

## Abstract

**Background:**

The variety of available flavors in e-cigarettes may be a driver for young people to start using these products. The objectives of our study were to examine the relationship between sensation-seeking behavior and e-cigarette use, and to identify the predictors of flavor use patterns among adolescents in Indonesia.

**Methods:**

Students aged 15 to 24 years participated from randomly selected high schools and universities in Indonesia. Participants answered questions about their demographic data, e-cigarette use, conventional cigarette use, and sensation-seeking. Flavor preferences were identified from eight different flavor categories. Multivariate multinomial regression analysis was employed to predict conventional cigarette and e-cigarette use among students. A latent class analysis was conducted to determine the number of latent classes of flavor use.

**Results:**

One thousand six hundred high school and university students, with a mean age of 18.2 years (SD 2.19), were recruited between March and August, 2023. Conventional cigarette use in the past 30 days was higher (16.3%) compared to e-cigarette use (13.3%, *p* = 0.017), with approximately 8.5% of students were being dual users. Higher levels of sensation-seeking significantly increased the odds of being a current e-cigarette user (OR = 2.54, 95%CI 1.99–3.25) and a current conventional cigarette smoker (OR = 2.38, 95%CI 1.85–3.07). Three groups of flavor classes were identified: 1) primarily menthol flavor users (14%), who had a strong association with current conventional cigarette use; 2) experimenters, who mostly preferred fruit-flavored e-cigarettes (76%); and 3) the multi-flavor user group (10%), who had a higher sensation-seeking tendency.

**Conclusions:**

Flavors, especially menthol and fruit flavors, attract youth, broaden the e-cigarette audience and are particularly appealing to high sensation-seekers. Banning these flavors could significantly deter e-cigarette initiation among youth.

## Background

The prevalence of electronic cigarette (e-cigarette) use is increasing worldwide, with adolescents and young adults particularly susceptible to this emerging trend, especially in high-income countries [1, 2]. Alongside the growing number of e-cigarette users, there has been an increase in the concurrent use of conventional cigarettes and e-cigarettes among adolescents. Estimates from the 2014–2019 Global Youth Tobacco Survey (GYTS) in 75 countries show that 4.6% of adolescents used both conventional and e-cigarettes simultaneously in the past 30 days [3].

Indonesia, a country with the highest rate of smoking among men in the world [4, 5], has seen a similar trend in e-cigarette use. Indonesia has just passed a new Health Law No. 17/2023, which includes general regulations on tobacco products, including e-cigarettes. A Government Regulation is needed to implement the law. The Indonesian government is currently in the process of drafting a detailed regulation that is a derivative of the law. Until this Government Regulation is officially issued, there are no specific regulations for e-cigarettes, except for fiscal regulations (imposition of excise taxes). The lack of regulation over the years likely contributed to a significant increase in e-cigarette use in Indonesia. According to a recent survey, the prevalence of e-cigarette use in Indonesia increased from 0.3% in 2011 to 3.0% in 2021 [6, 7]. E-cigarette use among adolescents aged 10–18 years also showed a significant increase within two years, rising from 1.2% in 2016 to 10.9% in 2018 [4, 8]. In 2019, a local study conducted among high school students in Jakarta found that 11.8% of the students used e-cigarettes during the last 30 days (current use), and more than half of current e-cigarette users (51.1%) also smoked conventional cigarettes in the past 30 days (dual use) [9].

The wide variety of e-cigarette flavors is often cited as playing a key role in vaping initiation, with flavors such as fruit and sweets being more likely to motivate young adults to start using e-cigarettes [10–13] and reduce the perception of harm associated with e-cigarettes [14]. A study in the United States in 2021 found that young adults generally preferred 'ice' flavored e-cigarettes, which was also associated with the increased use of conventional cigarettes and greater nicotine dependence [15]. It is estimated that there are currently more than 15,000 unique flavors of liquids for electronic vaping products [16]. A recent survey of e-cigarette retail stores in Indonesia found that all e-cigarette liquids sold in retail stores contained flavors, and the majority (92.6%) of product packaging featured cartoon or public figure images [17], which may be more appealing to adolescents and young adults.

Adolescence is a critical developmental period characterized by increased exploration, novelty seeking, and a tendency toward risk-taking behaviors [18]. In this period, adolescents with pronounced sensation-seeking traits are often attracted to new and intense experiences, which can influence their choices, including those related to substance use [19]. Sensation seeking is a personality trait characterized by the search for experiences and feelings that are varied, novel, complex, and intense, and by the willingness to take physical, social, legal, and financial risks for the sake of such experiences [20]. In a number of longitudinal and cross-sectional studies, sensation seeking has consistently predicted traditional cigarette smoking [21] and other risk behaviours, (e.g. alcohol consumption) [22], energy drink consumption [23], and risky driving [24]. By facilitating these behaviors, sensation-seeking behavior may have long-term effects on health and longevity, although we are not aware of any studies that have directly tested this hypothesis. The tendency of sensation-seeking can lead to engagement in risky behaviors, including smoking and experimenting with e-cigarettes, through several pathways [25]. First, it may increase the expectation of positive outcomes (such as taste and sensory experiences) associated with these behaviors. Secondly, it may reduce perceptions of potential risks or negative outcome expectations. Finally, it encourages the pursuit of stimulating environments where risky behaviors are shared and the formation and maintenance of relationships with peers who engage in such behaviors.

Recognizing sensation-seeking as a stable trait, less amenable to direct intervention [18], the focus of intervention should therefore shifts towards minimizing exposure to attractive stimuli – such as exciting flavors – that may encourage risky behaviors. Therefore, understanding the interplay between features of e-cigarettes and sensation seeking is crucial for comprehending the factors contributing to certain behaviors such as e-cigarette experimentation. This study explores the complex relationships between demographic variables, sensation-seeking, and the utilization of specific e-cigarette flavors among adolescents and young adults in Indonesia. We hypothesized that there is a strong correlation between sensation-seeking behavior and e-cigarette use, with a wide range of flavors being particularly appealing to adolescents and young adults who exhibit high levels of sensation-seeking traits.

## Methods

### Participants and procedure

This cross-sectional study included adolescents and young adults aged 15–24 years old from three provinces of Indonesia with the highest prevalence of e-cigarette users: Jakarta, Jogjakarta, and East Kalimantan [4]. Data collection was conducted from March to August 2023. Based on statistics from 2020–2021, the youth population (ages 15–24) was 1,689,230 in Jakarta, 600,100 in Jogjakarta, and 622,136 in East Kalimantan [26–28]. To accurately represent these populations in a study with a 95% confidence level and only 5% error, the required sample sizes were determined as 385 for Jakarta and 384 for both Jogjakarta and East Kalimantan. This means a total of 1,153 young people across these regions needed to be sampled for the study. To achieve the target quota, four high schools (two private and two public) and two universities (one private and one public) in each province were randomly selected from the available list provided by the Ministry of Education, Culture, Research, and Technology [29] and UniRank websites [30]. Direct mail and telephone communication were used to contact the principals of selected high schools and universities. After receiving permission, personal visits were made to schools and universities to distribute the questionnaire link in classrooms. To incentivize participation, 30 gifts worth IDR 50,000 (approx. USD 3) were provided to randomly selected respondents. All data were collected using the Qualtrics online platform. A total of 1,799 responses were received. All questionnaires containing responses to socio-demographic questions only (166) and disagreements with informed consent (33) were excluded. In total, responses from 1,600 participants were included in the analytic sample. Written informed consent was obtained from each participant. In the case of high school students, consent was also obtained from their respective classroom teachers.

### Measurements

This study employed a questionnaire to collect data from participants. In this report, we have used a total of nine variables: four pertaining to demographic information, three variables were related to cigarette and e-cigarette use behavior, one variable about e-cigarette liquid flavors that have been used, and the last one is the brief sensation seeking scale. We collected additional data related to expectations and perceived marketing, the analysis of which will be presented in another report due to space limitations. A two-step linguistic validation process was used to translate the questionnaire. The first step involved a forward–backward translation process (English-Bahasa) performed by two English-speaking Indonesian natives. The second step involved a cognitive interview with two high school students and two first-year university students from Indonesia. The authors evaluated and analyzed the results of the cognitive interviews and forward–backward translation process to adjust and finalize the questionnaire before conducting field data collection. It should be noted that while our research included a broader set of variables, this paper analyzes explicitly the variables mentioned, leaving the rest for later studies.

#### Patterns of cigarette smoking and e-cigarette use

To assess the use of conventional cigarette smoking and e-cigarette products, we adapted a questionnaire from a previous study [9]. Two items were used to assess the status of e-cigarette use. The first item asked participants if they had ever tried or experimented with e-cigarettes, even with just one or two puffs, with 'yes' and 'no' as response options. Participants who answered yes but responded that they did not use e-cigarettes in the past 30 days were categorized as ever e-cigarette users. Those who reported using e-cigarettes in the past 30 days were categorized as current users. A similar procedure was followed for conventional cigarettes. By conventional cigarettes we mean both kretek (clove) cigarettes and 'white' filtered cigarettes. In addition, those who reported using both conventional cigarettes and e-cigarettes alternately in the past 30 days were categorized as dual users.

#### Flavor

For e-cigarette users, we asked about the flavors of the e-cigarette liquid they used. The response categories for flavors were derived from the flavor wheel provided by the Dutch National Institute for Public Health and the Environment [31]. There were eight items related to flavors: menthol or mint, nuts, spices (e.g., clove, ginger, cinnamon), fruit, candy, beverages (coffee, tea), dessert, and tobacco flavor. The questions on flavor use only applied to those who reported trying or experimenting in the previous question (both current and former e-cigarette users).

#### Sensation Seeking Scale (SSS)

We used the 8-item Brief Sensation Seeking Scale (BSSS-8), which has been validated in many previous studies [32, 33]. Participants were instructed to respond using a five-point scale, ranging from "strongly disagree" to "strongly agree," to express their level of interest in various items reflecting experience seeking, thrill and adventure seeking, disinhibition, and boredom susceptibility. In this study, the average of the eight items formed the sensation seeking score. The internal consistency of the scale in this study is satisfactory (α = 0.81).

#### Demographics

Demographic information including students' age, sex, school/university location (rural or urban), and school/university type (private or public) were also used in this report.

### Statistical analysis

Initially, we computed descriptive statistics for all variables in our study. This was followed by a multivariate multinomial regression analysis to predict both current and ever use of conventional cigarette and e-cigarette among students, considering factors such as age, sex, school/university location (urban and rural), type (public and private), and sensation-seeking. Additionally, we explored correlations between flavor use and demographic, personality, and behavioral factors among current and ever e-cigarette users. Furthermore, we conducted a latent class analysis to gain deeper insights into flavor preference patterns among current and ever users. This analysis, guided by Collins & Lanza [34] and Vermunt & Magidson [35], aimed to identify subtypes of individuals (latent classes) with similar flavor use patterns. The determination of the number of latent classes involved the Bayesian information criteria parsimony index, entropy, solution replicability, and cluster interpretability. We also employed the Lo-Mendell-Rubin adjusted likelihood-ratio test (LRT) for final class determination, as described by Muthén & Muthén [36], where a low *p*-value (*p* < 0.05) favored the model with more classes. Furthermore, we compared the identified latent classes across various factors like age, sex, usage patterns, and sensation-seeking using DCon and DCat procedures [37]. The R3Step method, as implemented in the Mplus program, was applied for latent class regression analysis to predict group membership through a multinomial regression model [37]. All analyses were performed using SPSS version 26 and MPlus version 8.10.

## Result

### Descriptive statistics

Table 1 presents the descriptive statistics of our sample. Among the participants, a higher percentage reported currently smoking conventional cigarettes (16.3% [95% CI 14.5–18.2]) compared to those currently using e-cigarettes (13.3% [95% CI 11.6–14.9]; z = 2.39, *p* = 0.017). Dual use of both conventional cigarettes and e-cigarettes was reported by 8.5% [95% CI 7.1–9.9] of participants.

**Table 1:** Descriptive statistics and sex difference

Characteristics	Total sample	Sex		
	*N* = 1600	**Males** *N* = 602 (39.2%)	**Females** *N* = 935 (60.8%)	**χ**^**2**^**/t (*p*)**
Age, Mean (SD)	18.20 (2.19)	17.9 (2.31)	18.4 (2.10)	4.18 (*p* < .001)
Location N (%)				
Rural	319 (21.1)	129 (21.7)	180 (20.9)	0.12 (*p *= .731)
Urban	1195 (78.9)	466 (78.3)	680 (79.1)	
School/University Type N (%)				
Private	783 (48.9)	309 (51.3)	445 (47.6)	2.05 (*p *= 0.153)
Public	817 (51.1)	293 (48.7)	490 (52.4)	
Conventional Cigarette use N (%)				
Never smoker	1050 (65.6)	229 (38.0)	775 (82.9)	360.6 (*p* < .001)
Ever smoker^a^	289 (18.1)	161 (26.7)	117 (12.5)	
Current smoker^b^	261 (16.3)	212 (35.2)	43 (4.6)	
E-cigarette use N (%)				
Never user	1010 (63.4)	263 (44.1)_a_	696 (74.7)_b_	160.4 (*p* < .001)
Ever user^a^	371 (23.3)	192 (32.2)_a_	172 (18.5)_b_	
Current user^b^	211 (13.3)	142 (23.8)_a_	64 (6.9)_b_	
Current dual user N (%)	135 (8.5)	101 (16.9)	30 (3.2)	87.2 (*p* < .001)
E-cigarette flavor use N (%) (*N* = 582)				
Fruits flavors	340 (58.4)	184 (55.1)	150 (63.6)	4.09 (*p* = .043)
Menthol/mint flavors	243 (41.8)	156 (46.7)	84 (35.6)	7.00 (*p* = .008)
Candy flavors	142 (24.4)	75 (22.5)	63 (26.7)	1.36 (*p* = .244)
Dessert flavors	117 (20.1)	67 (20.1)	48 (20.3)	0.01 (*p* = .935
Beverages (coffee & tea) flavors	111 (19.1)	71 (21.3)	38 (16.1)	2.38 (*p* = .123
Nuts flavors	15 (2.6)	14 (4.2)	1 (0.4)	7.66 (*p* = .006)
Tobacco flavors	10 (1.7)	9 (2.7)	1 (0.4)	4.14 (*p* = .042)
Spices flavors	9 (1.5)	8 (2.4)	1 (0.4)	3.46 (*p* = .063
Other flavor	52 (8.9)	27 (8.1)	23 (9.7)	0.48 (*p* = .490)
No flavor use	6 (1.0)	4 (1.2)	2 (0.8)	0.16 (*p* = .687)
Number of flavors Mean (SD) [range]	1.78 (1.18) [0–7]	1.83 (1.29)	1.73 (1.04)	0.98 (*p *= 0.327)
BSSS-8^c^ Mean (SD)	2.53 (0.74)	2.63 (0.77)	2.48 (0.71)	t = 3.93 (*p* < .001)

Note: Proportions with different subscripts indicate statistically significant differences at the *p* < .05 level

^a^Ever used products but not in the past 30 days

^b^Used the products in the past 30 days

^c^The 8-item Brief Sensation Seeking Scale

A significant sex disparity was observed: boys reported higher rates of both current (23.8% vs. 6.9%) and ever use of e-cigarettes (32.2% vs. 18.5%) compared to girls. Boys were also more likely to report dual use (OR = 6.122 [4.01–9.34]). Fruit, menthol, and candy were the most popular flavors among e-cigarette users. Nearly half (*N* = 244, 41.9%) reported using multiple flavors. Sex differences emerged in flavor preferences; boys more commonly used both fruit and menthol flavors, while girls predominantly used fruit flavoring at a significantly higher rate.

### Predictors of e-cigarette and conventional cigarette use: demographic variables and sensation seeking

To analyze the factors influencing e-cigarette and conventional cigarette use, two multinomial logistic regression analyses were employed and compared to identify unique and overlapping determinants in the usage patterns of both types of cigarettes. The results are reported in Table 2. The likelihood of being a current e-cigarette user is increasing with higher age. Girls demonstrated a significantly lower likelihood of both current and ever e-cigarette use compared to boys. Additionally, students with higher levels of sensation-seeking significantly increased the odds of being current and ever e-cigarette users, even after controlling for sex.

**Table 2:** Multivariate multinomial regression model to predict user status (*N* = 1,349)

	**E-cigarette**		**Conventional cigarette**		**ΔOR** **Wald test (p)**
	OR	95% CI	OR	95% CI	
**Current use**					
Sensation seeking (BSSS-8)	2.54^***^	1.99–3.25	2.38^***^	1.85–3.07	0.23 (0.628)
Age	1.12^**^	1.04–1.21	1.34^***^	1.23–1.45	17.96 (*p* < .001)
Sex (Ref.: Male)	0.17^***^	0.12–0.25	0.05^***^	0.04–0.08	34.58 (*p* < .001)
Location (Ref.: Rural)	0.97	0.63–1.49	0.50^**^	0.33–0.76	10.10 (.002)
School type (Ref.: Private)	0.72	0.50–1.02	1.22	0.86–1.72	8.58 (.003)
**Ever use**					
Sensation seeking (BSSS-8)	1.80^***^	1.48–2.18	1.97^***^	1.59–2.42	0.66 (.417)
Age	1.01	0.95–1.08	1.07	0.99–1.15	1.84 (.176)
Sex (Ref.: Male)	0.36^***^	0.27–0.46	0.23^***^	0.17–0.30	7.66 (.006)
Location (Ref.: Rural)	0.73	0.53–1.00	0.80	0.56–1.16	0.23 (.628)
School type (Ref.: Private)	1.10	0.84–1.44	1.08	0.80–1.46	0.01 (.918)

*Ref.* Reference Group

The reference category is non-user/non-experimenter. ^***^: *p* < .001 ^**^: *p* < .005

Similar to e-cigarette use, the probability of being a current user of conventional cigarettes also increases with age. Compared to boys, girls were also less likely to be current or ever smokers of conventional cigarettes. Additionally, students with higher levels of sensation-seeking significantly increased odds of both being current and ever smokers of conventional cigarettes, even after controlling for sex. Furthermore, students in rural areas were more likely to be current conventional cigarette smokers than their urban counterparts, even after controlling for the individual explanatory factors we measured.

The comparison of odds ratios for the current use of e-cigarettes and conventional cigarettes revealed significant differences in age, sex, location, and type of school; however, as for school type, none of the odds ratios were different from 1.00. These analyses indicated that the relationship between these variables and e-cigarette use differs from that of conventional cigarette use. Specifically, the odds ratios for age are smaller for e-cigarettes, meaning that e-cigarette use is less dependent on age compared to conventional cigarettes. We also see a different pattern for location, with location explaining current conventional cigarette use but not e-cigarette use. However, after controlling for other variables, the association of sensation-seeking with both current e-cigarette and conventional cigarette use yields marginally varied odds ratios, but these are not statistically significantly different.

Despite all the marginal differences in odds ratios, no significant difference was found between the explanatory variables for ever use of e-cigarettes and conventional cigarettes, with the exception of sex, where a larger sex difference is found in relation to previous use of conventional cigarettes compared to previous use of e-cigarettes.

### Correlates of individual taste preference

Table 3 shows the correlation matrix between the use of different e-cigarette flavors and the explanatory variables among current and ever users. Age shows a weak but significant correlation with the use of fruit and multiple flavors. In terms of sex, there is a negative correlation with the use of menthol/mint, nuts, and tobacco flavors, indicating that boys use more than girls. Sensation-seeking is significantly associated with the preference for menthol/mint, dessert, and multiple flavor preference. Patterns of flavor preference vary by type of use: current e-cigarette users primarily prefer menthol/mint, fruit, and dessert flavors; conventional cigarette users who use or have used e-cigarette tend to choose menthol/mint, beverage, dessert, and tobacco flavors; and dual users (those who currently use both e-cigarettes and conventional cigarettes) show a preference for menthol/mint, dessert, and tobacco flavors. The overall weak strength of these correlations suggests the need to consider additional explanatory variables that may influence flavor preferences.

**Table 3:** Correlations between flavor use and demographic, personality, and behavioral factors among current and ever e-cigarette users

	Age	2	3	4	5	6	7	8	9	10	11	12	13	14
2. Sex	.07													
3. Sensation seeking	.02	-.09^*^												
4. Conventional Smoking	.16^**^	-.31^**^	.14^**^											
5. E-cigarette use	.13^**^	-.14^**^	.12^**^	.45^**^										
6. Dual-use	.15^**^	-.17^**^	.13^**^	.75^**^	.73^**^									
Flavors														
7. Menthol/Mint	-.02	-.12^**^	.09^*^	.17^**^	.16^**^	.12^**^								
8. Nuts	.00	-.12^**^	.05	.06	-.01	.04	.08^*^							
9. Spices	.05	-.08	.03	.05	.02	.06	-.02	.07						
10. Fruits	.10^*^	.07	.06	-.04	.09^*^	.00	-.02	.07	-.01					
11. Candy	-.02	.06	.01	-.01	.00	-.02	.05	.14^**^	.06	.07				
12. Beverages (coffee & tea)	-.02	-.06	.06	.08^*^	.04	.04	.15^**^	.14^**^	.08	.09^*^	.15^**^			
13. Dessert	.04	.00	.16^**^	.13^**^	.11^**^	.12^**^	.06	.19^**^	.04	.12^**^	.12^**^	.23^**^		
14. Tobacco	-.01	-.09^*^	.04	.10^*^	.07	.08^*^	.08	.15^**^	.19^**^	.00	.02	.10^*^	.10^*^	
15. Multiple flavours^a^	.09^*^	.05	.11^*^	.07	.14^**^	.06	.40^**^	.17^**^	.06	.45^**^	.38^**^	.38^**^	.38^**^	.13^*^

*N* = 532–582. ^*^: *p* < .05; ^**^: *p* < .01. Sex codes: 1 = Boys, 2 = Girls. Flavor is coded as 0 (not used) and 1 (used). The coefficients presented in bold are significant, at least at the *p* < .05 level

^a^The coding for multiple flavors is as follows: a single flavor is coded by '0', while the selection of more than one flavor is coded by '1'

### Patterns of flavor use

**Fig. 1:**
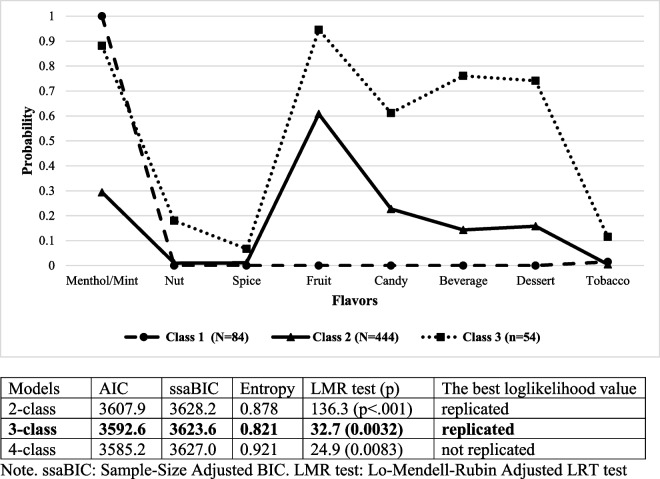
Latent classes of the pattern of flavors used in e-cigarettes

We conducted a latent class analysis using eight different e-cigarette flavors to identify distinct groups based on flavor preferences. Our analysis evaluated solutions ranging from one to four classes. The results, including information-based criteria and entropy for each solution, are shown in Fig. 1. We observed that the Akaike Information Criterion (AIC) decreased with the addition of more latent classes, whereas the sample size adjusted Bayesian Information Criterion (BIC) had the minimum value in the case of the three-class solution. The 4-class solution was not identified in the replications of the model testing. Therefore, the 3-class solution was retained.

As shown in Fig. 1, the largest proportion of respondents (76%) fell into class 2 which is characterized primarily by a preference for fruit-flavored e-cigarettes and a lower likelihood of using other flavors. Class 1, comprising 14% of respondents, predominantly favored menthol or mint flavors. The smallest group, class 3, consisted of users with a variety of flavor preferences, commonly choosing fruit, candy, beverage, dessert, and menthol flavors.

To further understand the characteristics of each latent class, we compared them across age, sex, pattern of use, and sensation-seeking. These comparisons were made using multinomial regression analysis with the R3Step method, accounting for the probabilistic nature of group membership [37]. The results are summarized in Table 4.

**Table 4:** Comparison of latent classes of e-cigarette flavor use

	Class 1 Only menthol/mint user *N* = 84	Class 2 Mainly fruit *N* = 444	Class 3 Multiple flavor users *N* = 54	Wald test (p)
Group comparisons^a^				
Age, Mean (SE)	17.74 (0.28)	18.33 (0.10)	17.90 (0.28)	5.21 (.074)
Sex % Boys	83.5%_a_	53.4%_b_	76.3%_a_	13.07 (< .001)
Current e-cigarette use	44.6%_ab_	32.8%_a_	53.5%_b_	5.53 (.063)
Current conventional cigarette use	61.8%_a_	28.9%_b_	66.8%_a_	21.61 (< .001)
Current dual use	33.3%_ab_	19.9%_a_	42.6%_b_	7.49 (0.024)
Sensation seeking Mean (SE)	2.77 (0.10)_a_	2.73 (0.04)_a_	3.12 (0.10)_b_	13.53 (< .001)
Multinomial regression model^b^				
Age, OR [95% CI]	0.91 [0.77–1.08]	Ref.	0.87 [0.71–1.07]	N/A
Sex (Ref.: Boys), OR [95% CI]	0.39 [0.11–1.33]		0.55 [0.25–1.21]	
Current e-cigarette use (Ref.: Ever use), OR [95% CI]	0.61 [0.22–1.70]		0.86 [0.34–2.18]	
Current conventional cigarette use (Ref.: No use), OR [95% CI]	3.58^*^ [1.28–9.97]		4.24^**^ [1.50–11.94]	
Sensation seeking Mean (SE), OR [95% CI]	1.05 [0.64–1.73]		1.83^*^ [1.03–3.24]	

Note: Proportions or means with different subscripts indicate statistically significant differences at the *p* < .05 level

*OR* Odds Ratio, *CI* Confidence Interval, *Ref.* Reference Group

^*^: *p* < .05; ^**^: *p* < .01

^a^DCON with continuous variable and DCAT with categorical variables were used

^b^Model is estimated with R3Step procedure [37]

The group primarily using menthol flavors (class 1) is predominantly composed of boys (83.5%) and shows a strong correlation with current conventional cigarette use. On the other hand, the group that prefers fruit flavors (Class 2) is less likely to include current users of both e-cigarettes and conventional cigarettes compared to other classes. The multi-flavor user group (Class 3) is characterized by a higher level of sensation-seeking. Similar to class 1, this group also has a higher proportion of boys and higher rates of current use of e-cigarettes, conventional cigarettes and dual use. Notably, the multinomial regression analysis showed that the odds of current conventional cigarette use is significantly higher in this multi-flavor user group (class 3) when compared with the reference group (class 2).

## Discussion

Our study examined e-cigarette and conventional cigarette use among Indonesian adolescents, with a novel focus on how e-cigarette flavors relate to use patterns and other explanatory variables. This approach offers a relatively new perspective for understanding these trends, not only by highlighting key drivers of adolescent tobacco use but also by unraveling the multiple roles of flavored e-cigarettes.

Both conventional and e-cigarette users in this study were predominantly male. This phenomenon is not surprising for conventional cigarettes, given that Indonesia has the highest male smoking rate in the world [4, 5]. However, as reflected in our study, e-cigarettes seem to be more accepted among females, although male users remain more dominant. The 2018 National Basic Health Research Survey showed that the proportion of e-cigarette use among females aged > 10 years was reported at 2.7%, which was only slightly lower than that of males at 2.8% [4]. In contrast to conventional cigarettes, which are often perceived as a masculine male identity [38, 39], e-cigarettes are perceived as less harmful and associated with a modern lifestyle [40–42], which may be more acceptable across gender. A recent study of e-cigarette promotional content on Instagram in Indonesia found that 64% of the content featured images of young women [43]. While this content may be targeted at men, it also contributes to the normalization of e-cigarette use among women. Further research in gender studies might be needed to better understand how these products are perceived.

Our study found that conventional cigarette use was more prevalent in rural areas than urban areas. This difference is probably due to the popularity of kretek cigarettes among Indonesians. Notably, Indonesian tobacco market is still dominated by kretek cigarettes, which is a traditional clove-flavored cigarette [44]. According to the 2018 National Health Survey, kretek cigarettes are more prevalent in rural areas (70.1%) than urban areas (65.8%). Conversely, filtered 'white' cigarettes and e-cigarettes have higher prevalence rates in urban areas (46.9% and 3.8%, respectively) and with rural areas (39.3% and 1.6%, respectively) [4]. 'White' cigarettes, as they are called in Indonesia, are typically produced by foreign companies [45], although since 2009, almost all major kretek companies have been bought by foreign tobacco companies [46]. Although the samples cannot be considered representative, current conventional cigarette use was still higher than e-cigarette use in both rural and urban groups (rural: 20.7% and 12.2%; urban: 16.1% and 14.2%, respectively).

Our findings suggest that e-cigarette use is less influenced by age than conventional cigarettes, implying that e-cigarette products are more appealing to younger age groups than conventional cigarettes. In addition to factors such as the wide availability of flavors and attractive packaging [17], the extensive promotion of e-cigarettes through online platforms [47–49] and the influence of digital culture among youth [50, 51] may also play a role in shaping this trend [52].

The study found three distinct groups of users based on their e-cigarette flavor preferences. First, the largest group, comprising 76% of respondents, primarily preferred fruit-flavored e-cigarettes. We can refer to this group as the experimenter users as it was less likely to include current users of both e-cigarettes and conventional cigarettes compared to other classes. This finding is in line with a previous study, where fruit flavor was reported as a stronger motivator for young adults aged 18–24 years to start experimenting with e-cigarettes than for adults aged 35–44 years [10]. The tobacco industry has a long history of using flavors in tobacco products, primarily as a strategy to attract youth [53, 54].

Second, the smaller group almost exclusively preferred the menthol or mint flavors (14%), had a high proportion of boys and was strongly associated with current conventional cigarette use. Individuals who use e-cigarette flavors, mainly mint/menthol and non-tobacco flavors, were more likely to report increased satisfaction with smoking e-cigarettes and have higher odds of perceived addiction to e-cigarette use compared to respondents who do not use flavored e-cigarettes, an important consideration for smokers [10]. Previously, the removal of menthol from conventional cigarettes was reported to reduce the likelihood of smoking initiation among adolescents [55]. This precedent highlights the importance of flavoring, particularly menthol/mint flavors and other similar variants such as 'ice' flavors in these products [15]. Several countries, including Ethiopia, Senegal, Turkey, Moldova, and the European Union, have banned the use of menthol flavoring in conventional cigarettes [56]. For e-cigarettes, China, the world's largest producer of e-cigarettes in 2021, implemented a ban on e-cigarette flavors other than tobacco [57]. In Europe, there are countries like Finland, Hungary, Netherlands, Ukraine, Lithuania that also ban flavors other than tobacco. Two other countries, Denmark and Estonia, allowed only tobacco and menthol flavors on e-cigarettes [58].

The third group had diverse flavor preferences, including fruit, candy, beverage, dessert and menthol was strongly associated with higher sensation-seeking tendencies. Sensation seekers tend to use multiple flavors. The wide variety of e-cigarette flavors can provide a unique and novel sensation, in line with their preference for exciting and stimulating experiences. Flavors are also seen as a potential positive reinforcement expectancy, and an expected higher hedonic reward—to which young people with high sensation seeking are particularly sensitive [25]. Thus, both flavors and their variety are potential motivational mechanisms that make e-cigarettes even more appealing to young people especially those with higher sensation seeking. Knowing that sensation-seeking, reward-seeking and novelty-seeking are characteristics of adolescents [25], product development that emphasizes flavors and their variety is a clear example of tobacco marketing to youth [59] in countries where such restrictions do not exist.

Consistent with our study's findings, numerous prior studies have established a strong correlation between high sensation seeking and the use of both conventional cigarettes and e-cigarettes among adolescents and young adults [60–63]. High sensation seeking is a common trait in this age group due to their developmental stage, biological changes, and identity exploration, with its peak occurring during adolescence and gradual decline in adulthood [18, 19]. Therefore, behaviors formed during this period, including participation in risky activities such as tobacco use, hold significant importance.

Aside from addiction problem, the use of nicotine during adolescence and youth can notably impact brain development, particularly in the prefrontal cortex, which responsible for decision-making and impulse control [64–66]. This issue is compounded when nicotine concentrations in consumed products are exceptionally high, as evidenced in a study of Indonesian retailers where some e-cigarette liquids contained nicotine levels as high as 50 mg [17]. The enduring consequences of nicotine exposure during this critical period underscore the imperative for prevention and early intervention to protect the developing brains of young individuals from these adverse effects.

As the Indonesian government is currently drafting government regulations as a derivative of the new Health Law, there is an opportunity to protect young people by including provisions to ban flavors and set a maximum level of nicotine in e-cigarette liquids. Governments worldwide, not just in Indonesia, should urgently implement the prohibition of various e-cigarette flavors as a strategic preventive measure if they have not already done so. Such a ban aims to deter young individuals from initiating health-compromising behaviors associated with e-cigarette use and potentially transitioning to conventional cigarette use.

### Study limitations

While the cross-sectional design of this study significantly limits causal inferences, it is presumed in our research that the explanatory variables, including demographic and personality factors, precede therefore, may predict behavior. However, it is also important to acknowledge that we cannot entirely rule out the presence of unmeasured third variables. Additionally, we cannot discount the possibility that the intake of nicotine through traditional and e-cigarette use may induce changes in sensation-seeking behavior.

In addition, the study sample was drawn from three Indonesian provinces with the highest prevalence of e-cigarette users in Indonesia, which may limit the generalizability of the findings to other regions. However, the unique cultural and regulatory context of Indonesia adds a layer of interest and relevance to this study. Data collection relied on self-reported responses, which may introduce recall bias and social desirability bias. Longitudinal studies with a representative sample would be needed to explore the temporal sequence and causal relationship between sensation-seeking, demographic factors, and e-cigarette use.

## Conclusions

Our findings highlight how a wide variety of flavors is particularly appealing to adolescents and young adults who exhibit high sensation-seeking traits. Given the stable nature of sensation-seeking behavior in this population, implementing a ban on flavored e-cigarettes emerges as a potentially highly effective intervention to reduce e-cigarette initiation. This approach recognizes the enduring nature of sensation-seeking behavior and aims to address the appeal that e-cigarette flavors have for this specific group. However, some flavors are not only interesting and appealing to young people with a high sensation-seeking but may also lead to a wider audience trying e-cigarettes. It may also make the product attractive to people who have not tried conventional cigarettes. Restricting the flavoring of e-cigarettes could be an important step in reducing the appeal of e-cigarettes and similar products to young people.

## Data Availability

The datasets utilized and/or examined in the present study can be obtained from the corresponding author upon reasonable request.

## Abbreviations

E-Cigarette: Electronic Cigarette
AIC: Akaike Information Criterion
BIC: Bayesian Information Criterion
LMR test: Lo-Mendell-Rubin test
LRT: Likelihood Ratio Test
